# Incidence and outcome characteristics of adverse event in surgery: an assessment based on systematic reviews of barbed suture

**DOI:** 10.1186/s12874-025-02607-0

**Published:** 2025-07-01

**Authors:** Xianlin Gu, Qiong Guo, Qingwen Zhao, Guiyu Jiang, Yanjiao Shen, Youlin Long, Xin Chen, Yifei Lin, Jin Huang, Liang Du

**Affiliations:** 1 Department of Urology, West China Hospital, Sichuan University, Chengdu, People’s Republic of China; 2 Innovation Institute for Integration of Medicine and Engineering, West China Hospital, Sichuan University, No. 37, Guoxue Xiang, Chengdu, Sichuan 610041 People’s Republic of China; 3 Chinese Evidence-Based Medicine Center, West China Hospital, Sichuan University, Chengdu, People’s Republic of China; 4 West China Medical Publishers, West China Hospital, Sichuan University, Chengdu, People’s Republic of China; 5 West China School of Public Health, Sichuan University, Chengdu, People’s Republic of China; 6 Department of Epidemiology and Health Statistics, West China School of Public Health and West China Fourth Hospital, Sichuan University, Chengdu, People’s Republic of China

**Keywords:** Adverse event, Systematic review, Meta-analysis, Barbed suture, Complications

## Abstract

**Background:**

Systematic reviews (SRs) have affirmed the efficacy of barbed sutures (BS), but raise safety concerns, particularly in adverse events (AEs). We conducted a cross-sectional study to comprehensively collate SRs on BS, summarize the incidence of AEs, and analyze the characteristics of the reported indicators.

**Methods:**

A thorough search was conducted in PubMed, Embase, China National Knowledge Infrastructure, Wanfang Database, China Biology Medicine disc and VIP Database from inception to April 14, 2025, and the methodological quality was evaluated using AMSTAR-2. AE characteristics of the SR were evaluated utilizing a self-developed item evaluation form.

**Results:**

A total of 52 SRs were included, encompassing 15 surgical procedures and 32 AEs. In terms of methodological quality, 1 SR was rated as high, 5 as moderate, 10 as low, and 36 as critically low. Notably, 92.0% of the SRs did not provide definitions for AEs, 76.0% did not consider severity grading, and none of the SRs assessed the adequacy of sample size. The use of BS significantly reduced the occurrence of needle pricks and biliary fistulas, in addition to increase the rates of suture breakage. Statistically insignificant results included wound dehiscence, blister, exudation, peri − incisional erythema, aseptic redness, hematoma, bleeding, urinary fistulae, stitch abscess, cellulitis, anastomotic stenosis and leak. Inconsistencies were observed in ecchymosis, vaginal cuff dehiscence, postoperative leakage rate after radical prostatectomy, infection, anastomotic bleeding, and overall postoperative complications across different SRs.

**Conclusion:**

Evidence from SRs has demonstrated advantages as well as certain disadvantages of BSs — such as an increase in the likelihood of suture breakage and wound-related complications. Current evidence remains inadequate and inconsistent, due to lack of definitions for AEs, severity grading, sample size assessments, and details on suture brand and location of use.

**Supplementary Information:**

The online version contains supplementary material available at 10.1186/s12874-025-02607-0.

## Introduction

Clinical practice guidelines (CPG) are essential for healthcare personnel to make efficient and high-quality clinical diagnostic and treatment decisions. Following the update to the definition of CPG by the Institute of Medicine in 2011, [1] systematic reviews (SR) have become an indispensable source of evidence for guideline development.

Recently, there has been a growing number of guidelines related to medical devices such as sutures. For instance, Faria et al. has developed an expert consensus on robot-assisted radical prostatectomy, [2] and barbed suture (BS), an innovative suture material that eliminates the need for knots, has been included in this consensus based on a SR [3]. Existing SRs have provided relatively clear assessments of the effectiveness of BS, demonstrating significant reductions in suture and operation times [4, 5]. However, there is no consensus on the safety assessment. Furthermore, the quality of SR can affect the scientific rigor of guideline development. Previous research has shown several deficiencies in the implementation, methodology, and reporting of published SRs, [6-8] with over one-third of them failing to provide a complete report on adverse event (AE) outcomes [9]. Hence, this study aims to analyze the current status of AE outcomes in SRs with BS as an intervention offering insights for future considerations of safety in the development and updates of guidelines/consensus statements on suture materials.

## Methods

### Development of items on the current status of AEs

Based on the FDA’s definition of AEs, we define AEs as any undesirable experience associated with the use of BSs in a patient [10]. In order to assess the current setting of AE outcome indicators in SRs, we followed the harms reporting consort statement developed for randomized controlled trials (RCTs) and SRs, [7-11] taking into account the key considerations in guideline development based on SRs, such as balancing evidence between benefits and harms and ensuring methodological quality. Comprehensive consideration of sample size and other aspects led to the formulation of a set of AE characteristics assessment items for SRs (Table S1), which were then applied in SRs involving BS as an intervention.

### Search strategy and study selection

PubMed, Embase (OVID), China National Knowledge Infrastructure (CNKI), Wanfang database, China Biology Medicine disc (CBM) and VIP database were searched for SRs of barbed suture as interventions from inception to April 14, 2025, using the following terms: (((“barbed” OR “knotless”) AND (“suture” OR “suturing”)) OR “V-loc” OR “Stratafix” OR “Quill”) AND (“systematic review” OR “meta-analysis”). See supplementary material for detailed search strategies (Appendix S1). Besides, reference list of included SRs was reviewed to identify potentially eligible studies.

After removing duplicate studies, two reviewers independently screened all titles and abstracts. Subsequently, full texts were downloaded for further screening. Any disagreements were resolved through consensus after discussion or consultation with a third reviewer.

### Eligibility criteria

Studies were included according to the following inclusion criteria: (a) study design: SRs and/or meta-analyses; (b) participants: patients underwent surgical operation; (c) intervention: barbed suture, without restriction on the types (i.e., bidirectional, unidirectional) or brands (i.e., QUILL, Stratafix, V-LOC); (d) comparation: non-barbed suture or other suture techniques.

The exclusion criteria were: (a) duplicate publication; (b) conference abstract; (c) non-English and non-Chinese; (d) no full text.

### Data extraction and quality assessment

Two reviewers independently performed the data extraction using a unified form. Any discrepancies were resolved by consulting a third reviewer. Extracted data included: (a) general characteristics of SRs: first author, country, year of publication, study type, number of included studies, sample size, surgical procedures, and follow-up time, etc.; (b) effect size indicators of AEs (e.g., odds ratio, OR; relative risk, RR; risk difference, RD; mean difference, MD*)*.

Besides, two reviewers independently assessed the methodological quality of the included SRs according to the AMSTAR-2 tool [12]. The AMSTAR-2 comprises 16 domains, considering 7 key items, each SR’s methodology quality was rated as “high”, “moderate”, “low” and “critically low”. Two reviewers cross checked the results and disagreements were resolved by consensus with a third reviewer.

## Results

**Fig. 1:**
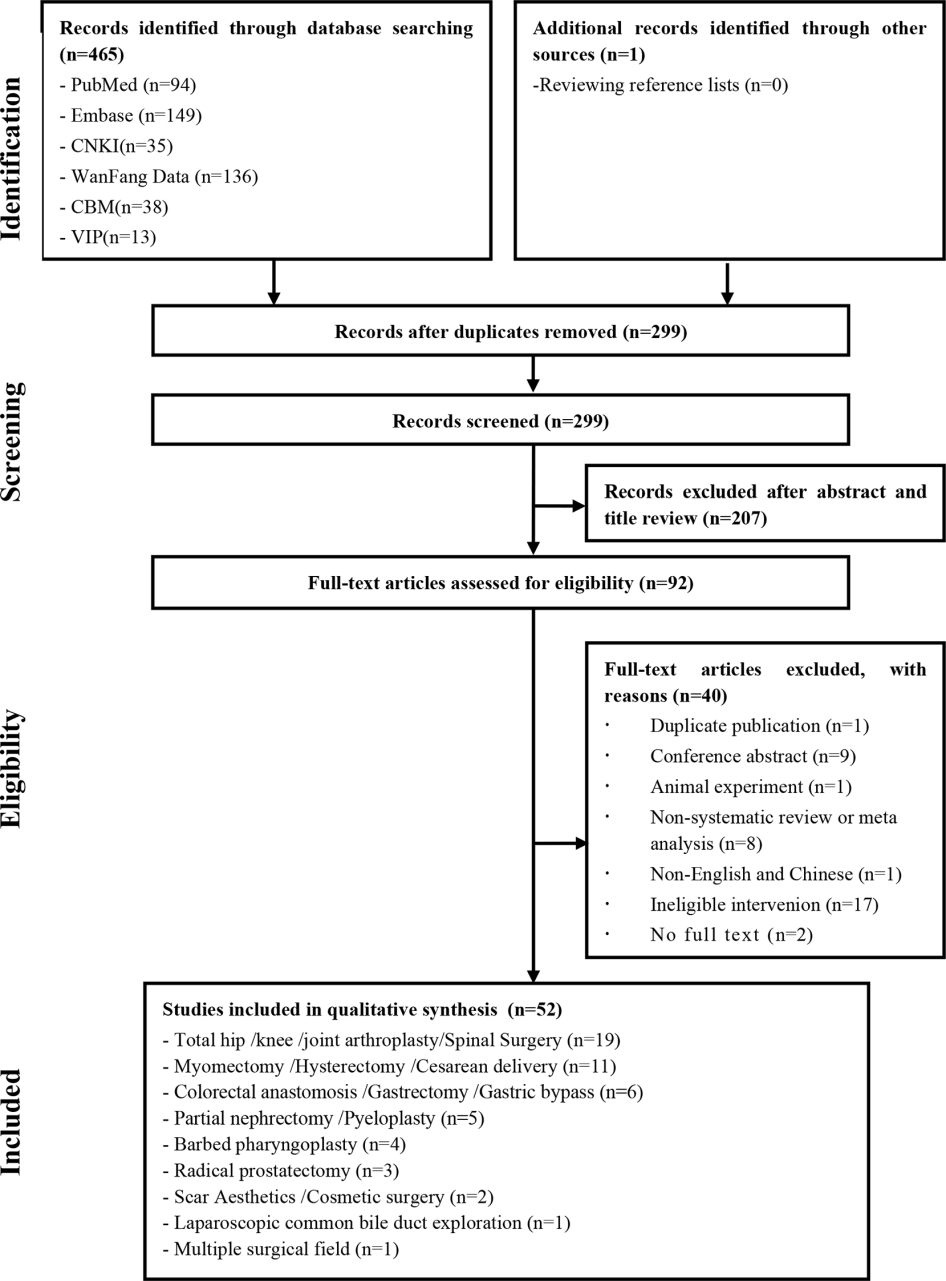
Flow chart diagram of SR selection

A total of 465 records were initially retrieved. Ultimately, 52 SRs involving 154 unique observational studies and 65 unique RCTs were included (Fig. 1). All included SRs were published after 2014, covering orthopaedic surgery [13-59] and cosmetic surgery [60-61]. (Table 1, Table S2) Additionally, one study covered various surgical specialties [62]. According to the AMSTAR-2, 36 studies were rated as critically low quality, nine as low quality, six as moderate quality, and one as high quality (Table 1, Figure S1, Table S3).

**Table 1:** Baseline characteristics of SRs

Characteristics	*n*	%
**1) Type of surgeries** (*n* = 40) (No. of RCTs/ No. of primary studies*)		
Total hip/ knee arthroplasty/ spinal Surgery	19 (22/49)	36.5 (44.9)
Cesarean delivery/hysterectomy/myomectomy	11 (20/63)	21.2 (31.7)
Gastrointestinal surgery	6 (4/18)	11.5 (22.2)
Barbed pharyngoplasty	4 (1/21)	7.7 (4.8)
Partial nephrectomy	4 (5/23)	7.7 (21.7)
Radical prostatectomy	3 (3/12)	5.8 (25.0)
Scar aesthetics**/cosmetic surgery	2 (5/15)	3.8 (33.3)
Minimally invasive pyeloplasty	1 (0/5)	1.9 (0.0)
Laparoscopic common bile duct exploration	1 (3/10)	1.9 (30.0)
Surgical field (involves a variety of surgeries)	1 (17/17)	1.9 (100.0)
**2) Publication year**		
2021 ~ 2025	19	36.5
2016 ~ 2020	26	50.0
2014 ~ 2015	7	13.5
**3) Country of first author**		
China	23	44.2
Italy	8	15.4
USA	5	9.6
Canada	3	5.8
India	3	5.8
Brazil	2	3.9
Greece	2	3.9
Other (Germany, Israel, Poland, Thailand, Tunisia, United Kindom)	6	11.5
**4) Language of full-text**		
Chinese	8	15.4
English	44	64.6
**5) Number of subjects included in a SR**		
Median (IQR)	1219.5 [758.3, 2271.5]	
<=500	9	17.3
500 ~ 1000	14	26.9
1000 ~ 5000	22	42.3
>=5000	7	13.5
**6) Number of primary studies included in a SR**		
Median (IQR)	9.0 [6.8, 12.0]	
4 ~ 10	27	67.5
11 ~ 17	13	32.5
**7) Type of included primary studies**		
only RCTs	12	23.1
only observational studies	4	7.7
RCTs + observational studies	36	69.2
**8) AMSTAR-2 assessment result**		
High	1	1.9
Moderate	5	9.6
Low	10	19.2
Critically low	36	69.2

*: Figures in parentheses indicate the total RCTs and primary studies for each surgical procedure, while percentages in parentheses show the proportion of RCTs among the total studies

**: This study evaluated postoperative scar aesthetic outcomes, involving total knee arthroplasties, abdominoplasties, multiple cosmetic surgeries, cesarean sections, gynecologic procedures, and abdominoplasties or reduction mammoplasty

### Characteristics of AEs in SR of BS

We used the developed items to evaluate the included SRs and assessed the characteristics of AEs (Table S4). Among the 52 SRs, two focused exclusively on efficacy outcomes, while 49 (94.2%) addressed both efficacy and AEs. Of the 50 SRs that included AEs, 16 (32.0%) reported only pooled postoperative complication rates without detailing individual AEs, and only 3 (6.0%) provided precise definitions for all included AEs. Additionally, 6 SRs (12.0%) classified AEs using self-defined grading criteria, and another 6 (12.0%) applied the Clavien-Dindo surgical complication grading system. Notably, only half of the SRs specified the time frame for AE surveillance (Table S3).

**Fig. 2:**
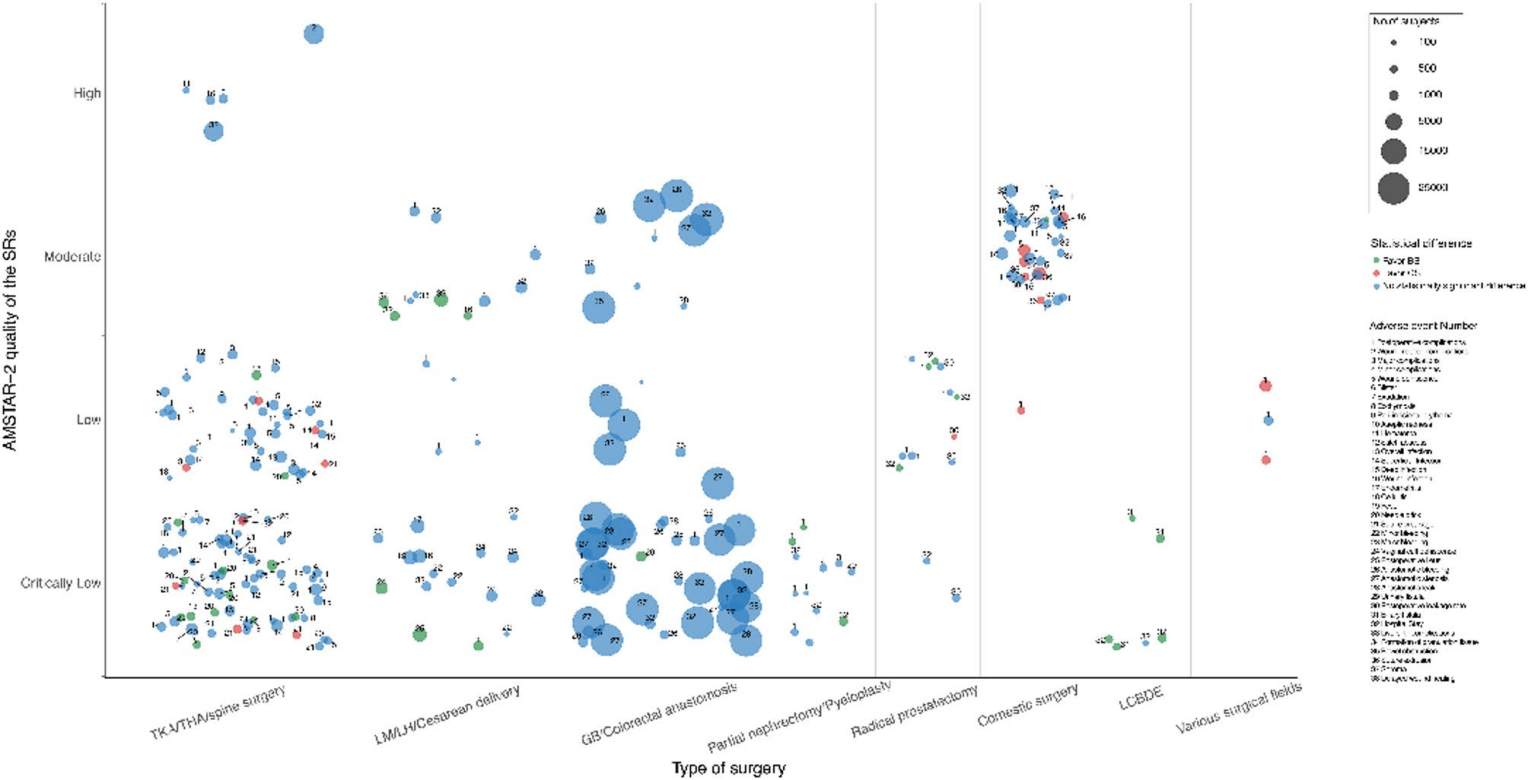
Bubble plot. Each bubble represents an AE, and the chart displays information from five dimensions: (1) the author’s conclusions are represented as “favor BS”, “favor CS”, and “no differential effect” using different bubble colors; (2) The y-axis is the quality assessment of AMSTAR-2; (3) The sample size of each AE is indicated by the bubble size; (4) Each block on the x-axis represents the classification of procedure types

**Fig. 3:**
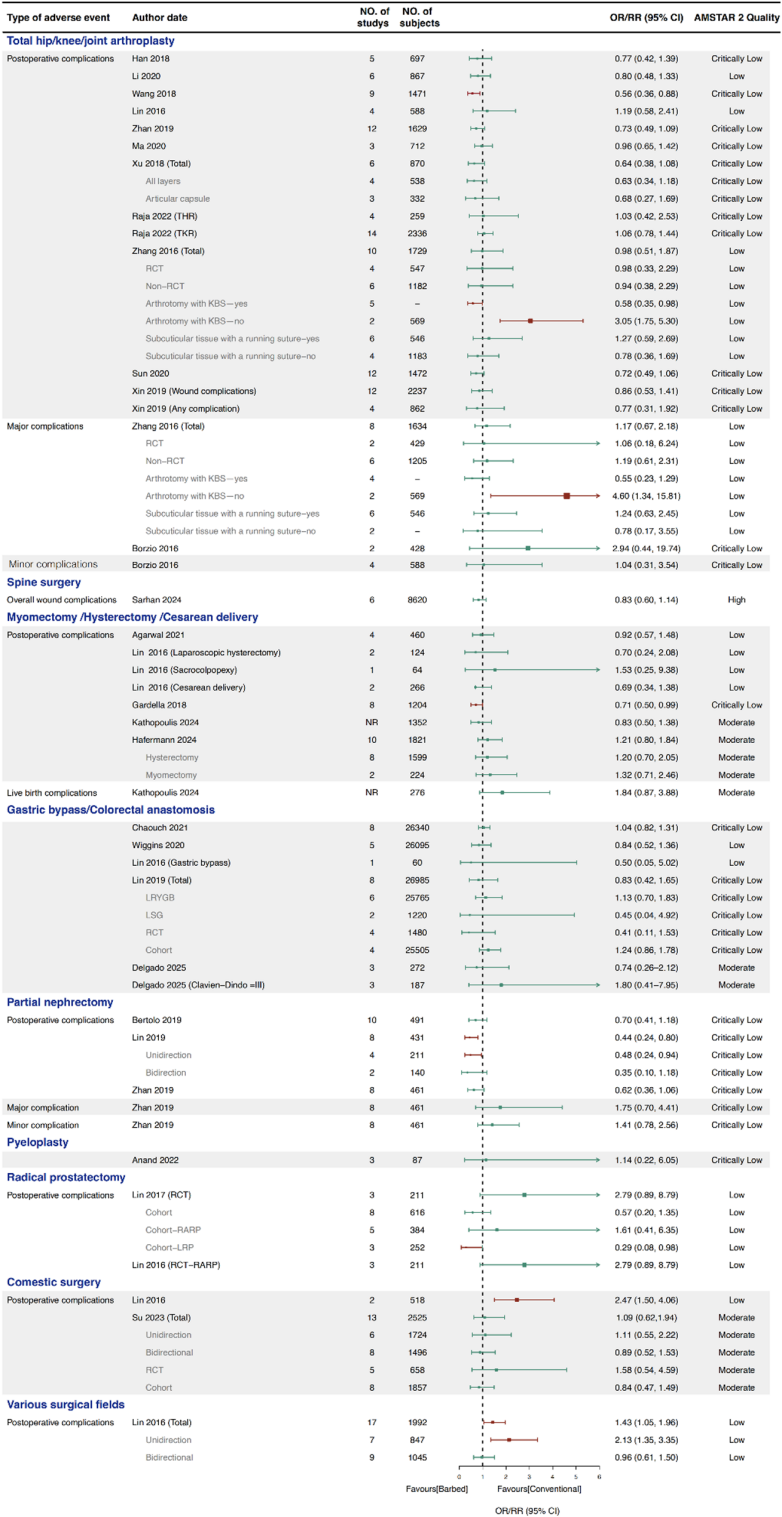
Forest plot of overall postoperative complications. (-: Unextractable data. *: Surgery included cesarean delivery, laparoscopic hysterectomy, laparoscopic myomectomy, robot-assisted laparoscopic prostatectomy, arthroplasty, gastric bypass, cosmetic surgery, and Sacrocolpopexy. Red lines indicate results with a statistically significant difference, and green lines indicate results without statistically significant difference, so as follows.)

**Fig. 4:**
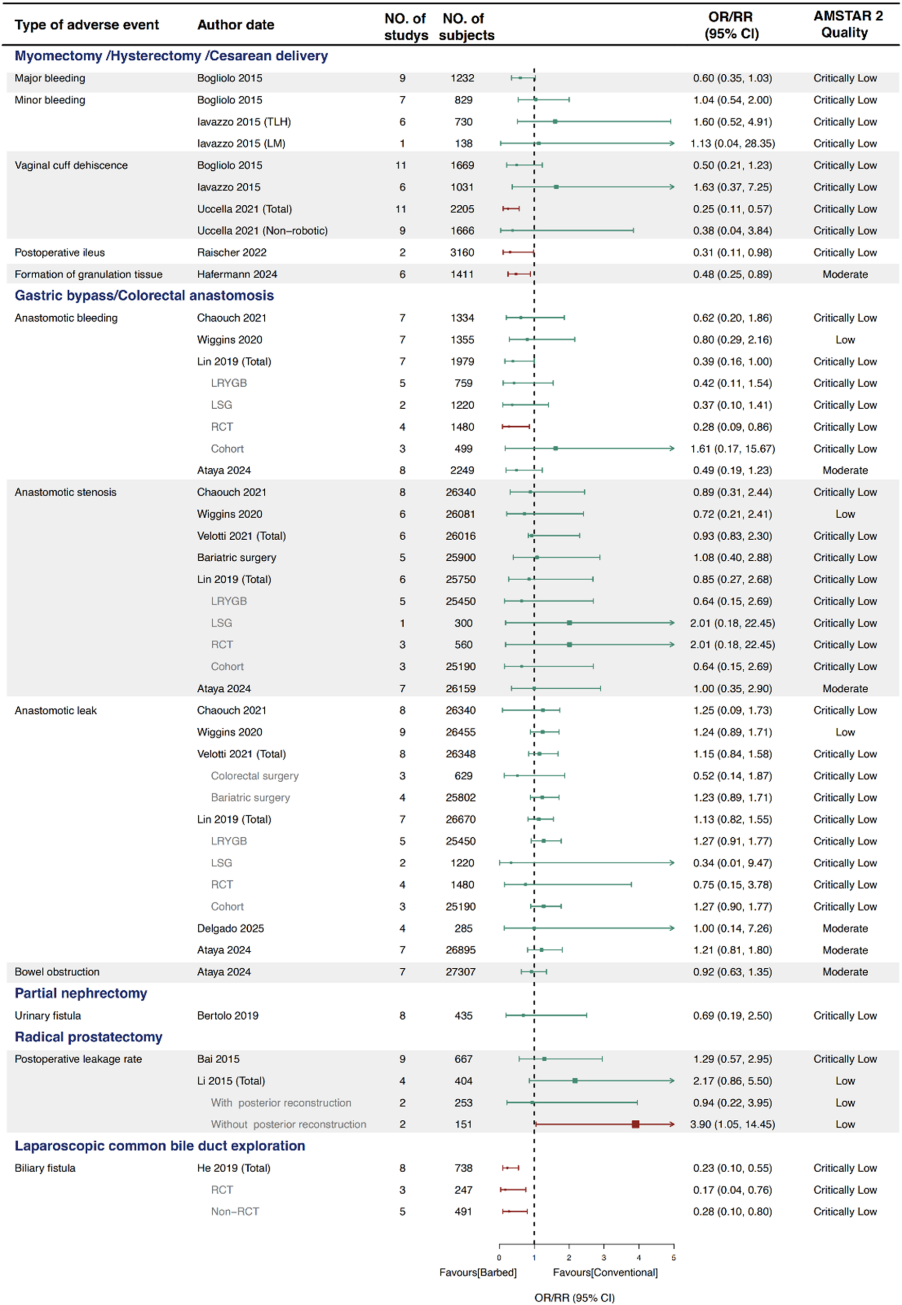
Forest plot of anastomotic site complications

**Fig. 5:**
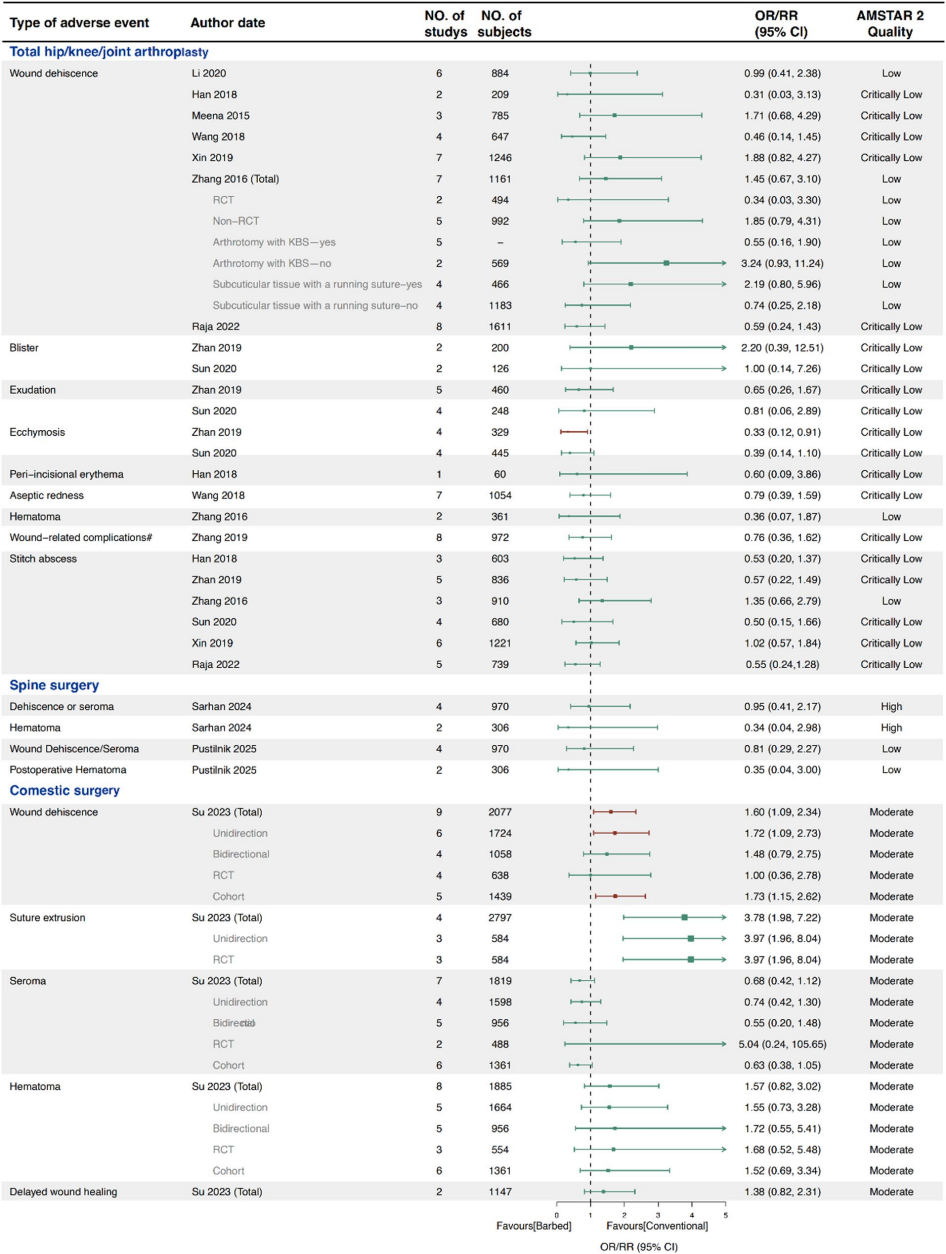
Forest plot of poor wound healing

The bubble chart (Fig. 2) showed the surgical fields, methodological quality, sample size, AEs involved and their statistical effects in the 50 SRs. According to the bubble size (Fig. 2), SRs with the most maximal sample sizes were predominantly focused on gastrointestinal surgery, each incorporating over 20,000 cases, and there was no significantly statistical difference in the results of AEs such as anastomotic stenosis and anastomotic leak, except for one subgroup that showed that the use of BS could significantly reduce the incidence of anastomotic bleeding. Other surgical areas included samples ranging from 120 to 3,332, and no studies assessed the adequacy of the sample size (Table S4). Notably, more than 50% of the statistically significant AEs above were results in subgroup analyses (Figs. 3, 4, 5 and 6).

## Summary of the clinical profile of AEs

### Overall postoperative complications

A total of 27 SRs conducted 73 meta-analyses of overall postoperative complications (Fig. 3). Across gastrointestinal surgery and radical prostatectomy, all meta-analyses reported no statistically significant differences between the BS and conventional suture groups. In total knee arthroplasty (TKA), total hip arthroplasty (THA), hysterectomy, myomectomy, cesarean delivery, and partial nephrectomy, most studies similarly found no significant differences in complication rates. However, three studies reported significantly fewer postoperative complications with BS in TKA [24] (OR 0.56, 95%CI 0.36 to 0.88), Partial nephrectomy [34] (OR 0.44, 95%CI 0.24 to 0.80) and laparoscopic myomectomy [43] (OR 0.71, 95%CI 0.50 to 0.99). Conversely, one SR [62] covering multiple surgical fields found higher complication rates in the BS group overall (OR 1.43, 95% CI 1.05 to 1.96), with a subgroup analysis of cosmetic surgery showing a significantly greater increase (OR 2.47, 95% CI 1.50 to 4.06).

Moreover, subgroup analyses were performed by suture type (whether the arthrotomy was closed with BS), suturing method (interrupted vs. running), study design (RCT vs. non-RCT), BS type (unidirectional vs. bidirectional), and surgery type (robotic vs. non-robotic, LRYGB vs. LSG, RARP vs. LRP, etc.). Notably, significant differences were observed in subgroups based on suture type [26], study design [35], and BS type [34, 62].

### Anastomosis site complications

A total of 15 SRs conducted 49 meta-analyses of anastomotic complications (Fig. 4). All meta-analyses reported no significant differences in anastomotic bleeding, stenosis, or leakage between gastrointestinal surgeries. Likewise, during partial nephrectomy and radical prostatectomy, all three meta-analyses found no significant differences in urinary leakage rates. Nevertheless, when referring to hysterectomy and myomectomy, one meta-analysis reported a significant reduction in vaginal cuff dehiscence with BS [41] (OR 0.25, 95% CI 0.11–0.57), and one study [40] observed a lower incidence of postoperative ileus in the BS group (OR 0.31, 95% CI 0.11–0.89), Additionally, a single SR focusing on primary choledochal closure showed that BS significantly reduced the risk of biliary fistula [55] (RR 0.23, 95% CI 0.10–0.55). Hafermann et al [39] also reported a reduced risk of granulation tissue formation following hysterectomy (RR 0.48, 95% CI 0.25–0.89).

Subgroup analyses were also conducted based on study design (RCT vs. non-RCT), surgery type (robotic vs. non-robotic, LRYGB vs. LSG, etc.), and surgical approach (with or without posterior reconstruction). Notably, one subgroup analysis of bariatric surgery found that [54], in RCTs, the incidence of anastomotic bleeding was significantly lower in the BS group (OR 0.28, 95% CI 0.09–0.86).

### Poor wound healing

A total of 13 SRs conducted 52 meta-analyses on poor wound healing outcomes following TKA, THA, spine surgery, and cosmetic surgery (Fig. 5). No significant differences were found between BS and conventional sutures for outcomes such as stitch abscess, blister, exudation, peri-incisional erythema, aseptic redness, hematoma, seroma, and delayed wound healing. However, one meta-analysis [21] reported a significant reduction in ecchymosis with BS (OR 0.33, 95% CI 0.12–0.91). In cosmetic surgery, BS was associated with a higher risk of wound dehiscence (OR 1.60, 95% CI 1.09–2.34) and suture extrusion [60] (OR 3.78, 95% CI 1.98–7.22).

Subgroup analyses based on suture type (whether the arthrotomy was closed with BS), suturing method (interrupted vs. running), and study design (RCT vs. non-RCT) were also performed. Significant differences were observed in subgroups based on study design and BS type [60].

### Infection

A total of 15 SRs conducted 32 meta-analyses on surgical site infection following TKA, THA, spine surgery, cesarean delivery, and cosmetic surgery (Fig. 6A). Zhang et al [19]. reported a significantly lower overall infection rate in the BS group with primary TKA/THA (RR 0.31, 95% CI 0.14–0.69). Similarly, Pustilnik et al [13] found a reduction in total infection after spine surgery (RR 0.55, 95% CI 0.31–0.98), In addition, one meta-analysis of hysterectomy reported a significantly lower infection rate with BS [39] (RR 0.26, 95% CI 0.09–0.78).

In addition, Zhang et al [26] conducted a subgroup analysis based on suture type (whether the arthrotomy was closed with BS) and reported a higher rate of superficial infection when BS was not used for arthrotomy closure (OR 3.85, 95% CI 1.71–8.67).

### Intraoperative suture issues

A total of 10 studies conducted 24 meta-analyses on intraoperative suture-related issues during TKA and THA (Fig. 6B). For needle pricks, nine meta-analyses reported that BS significantly reduced the incidence [15-30], while one [23] found no significant difference. Regarding suture breakage, four meta-analyses [24, 28, 4, 30] reported a significant increase with BS, whereas the others [16, 18, 21, 23, 28, 30] found no significant difference.

Xu et al [30]. performed subgroup analyses of needle prick and suture breakage based on the layers of surgical closure (full-thickness vs. articular capsule) and found differing results.

### Length of hospital stay

A total of 19 SRs conducted 33 meta-analyses on hospital stay (Fig. 6C). For laparoscopic myomectomy, gastrointestinal surgery, spine surgery, and cosmetic surgery, all meta-analyses reported no significant differences between BS and conventional suture groups. However, four SRs found significantly shorter hospital stays with BS in hysterectomy/myomectomy^40^ (MD -0.24, 95% CI -0.43 to -0.06), partial nephrectomy [37] (MD -0.35, 95% CI -0.69 to -0.11), primary choledochal closure [55] (MD -0.78, 95% CI -1.47 to -0.09, and radical prostatectomy [35] (MD -0.96, 95% CI -1.80 to -0.11). In contrast, Raja et al. reported a longer hospital stay with BS during TKA and THA (MD 0.29, 95% CI 0.06–0.53).

Subgroup analyses based on BS type (unidirectional vs. bidirectional), study design (RCT vs. non-RCT), and surgery type (LRYGB vs. LSG, RARP vs. LRP) were also performed. Notably, different results were found in subgroups based on study design [55] and BS type [60].

**Fg. 6i:**
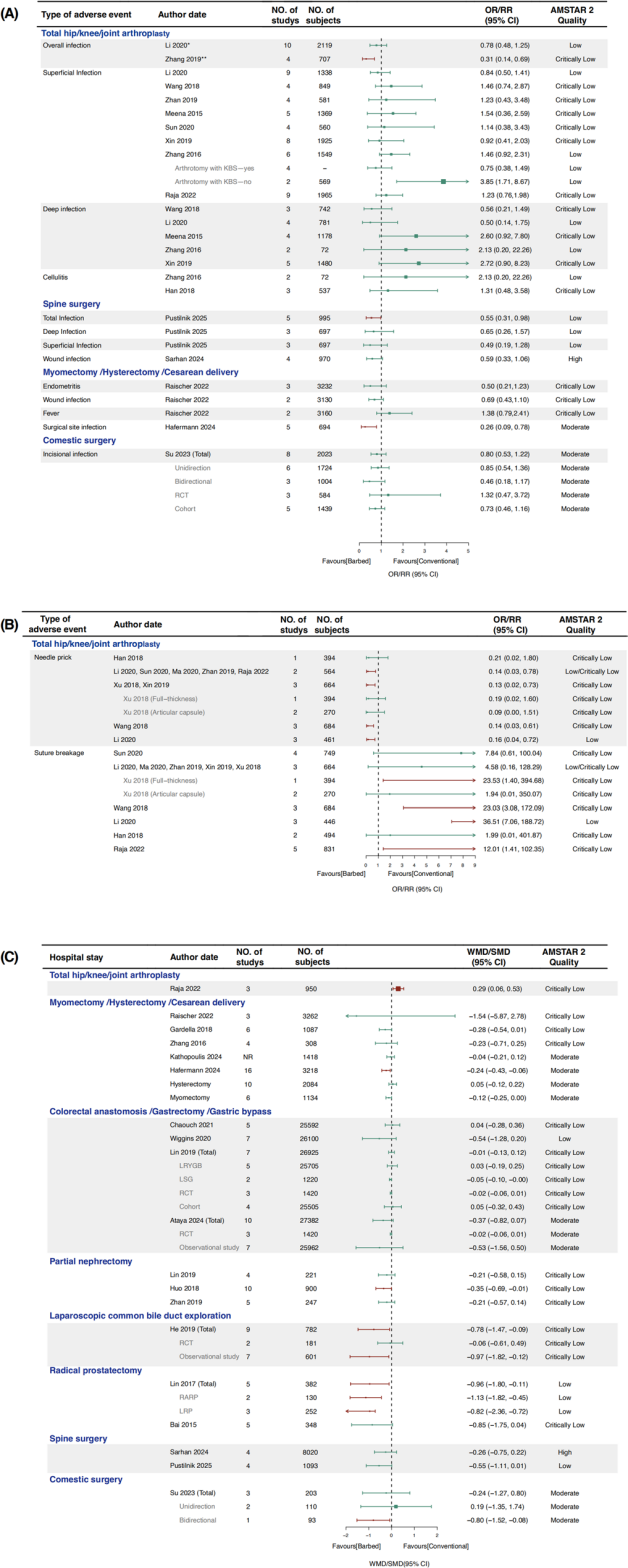
Forest plot of infection, intraoperative suture issues and hospital stay. **(A)** Infection (-: Unextractable data. *: Overall infection included superficial and deep infection. **: Overall infection included stitch abscess, superficial infection and deep infection.); **(B)** Intraoperative suture issues (*: SRs reporting the same meta-analysis results.) **(C)** Hospital stay

## Discussion

This study reviewed a total of 452 SRs of BS, conducted a systematic summary of the current research findings related to 32 different types of AEs associated with the use of BS in 15 surgical domains. Evidence from SRs has demonstrated advantages as well as certain disadvantages of BSs —such as an increase in the likelihood of suture breakage and wound-related complications, with 88.5% of the studies rated as having low or critically low methodological quality. The analysis covered areas such as their distribution within surgical domains, the characteristics of AEs, considerations of their severity, and the assessment of sample size adequacy. Notably, 92.0% of the SRs did not provide specific definitions for AEs, while 76.0% did not consider the grading of severity, and none of the SRs evaluated the adequacy of sample size.

This study identified a site-specific pattern in AEs associated with BS. BS use in fascia/wound closure was linked to a higher incidence of poor wound complications, while in anastomotic suturing, BS showed a reduced risk of complications. This may reflect differences in tissue environments: skin is superficial, under variable tension, and exposed to external forces, making it more susceptible to local irritation and infection from the barbed structure. In contrast, anastomotic sites are deep, well-vascularized, and benefit from the even tension and secure fixation provided by BS, which may lower the risk of leakage and related complications. These findings highlight the importance of tailoring suture selection to specific surgical contexts.

Although a substantial number of SRs on barbed sutures (BS) are currently available, their distribution across surgical fields remains uneven. The development of generalized guidelines is limited by the lack of evidence in certain surgical areas, highlighting the need for further research to strengthen the suture-related evidence base. In addition to the surgical types already explored in this study, many primary studies have been published clinically, including skin suturing after thoracic implantation, [63] scalp suturing following craniotomy for skull tumors, [64, 65] nasal suturing for treating cerebrospinal fluid leaks, [66] and suturing in lumbar [67] surgeries, for which there are currently no SRs.

The results of this study showed that 46 SRs did not provide definition for any AE, this could be attributed to potential space constraints, as some studies prioritize definitions related to effecacy indicators as their primary outcome measures. Another possibility is that the authors of SRs may not uniformly provide definitions for AEs. This ambiguity not only makes it difficult to use the information of SR directly and effectively, but also makes the consistency of AEs poor or difficult to judge, and the pooled results may be biased. Therefore, SRs should also fully consider the suture site, severity, duration, prognosis and other characteristics of AEs, and provide a comprehensive definition of each event. In the context of clinical and secondary studies, emphasis on the severity and predictability of AEs is paramount [68] Notably, most SRs overlooked the grading of AE severity, possibly due to a lack of severity-related information in primary studies or the SR authors’ oversight. The widely employed Clavien-Dindo classification system, introduced by Dindo et al., is a predominant grading system based on subsequent treatment requirements and the complexity of complications [69]. While alternative systems for classifying AE severity have been synthesized by scholars, [70, 71] their integration into clinical research has shown a gradual increase over time. Nevertheless, the citation rates remain modest, averaging two studies per year, [71] aligning with the outcomes of our investigation. It is plausible that SR authors may perceive these systems as presently unfit for their studies, underscoring the potential utility of developing tools for grading suture-related AEs to enhance the evaluation of suture safety. Furthermore, among the studies included, two SRs focused solely on specific AEs (e.g., vaginal cuff dehiscence), while others exploring all AEs reported only one indicator. For instance, in He et al.‘s study, only postoperative bile leakage was assessed among the indicator “postoperative complications”, reflecting a singular focus in the sole existing study on primary suturing of the common bile duct with barbed sutures. This limited scope raises concerns about potential oversight of unexpected adverse events.

In this study, over 50% of positive AEs were subgroup outcomes, emphasizing the potential impact of factors like suture level, BS type, and surgical approach. Attention to these subgroup outcomes is crucial in the practical clinical application of BS. In addition, over half of the included SRs did not report follow-up times, possibly obscuring long-term AEs related to suture. Considering the degradable nature of the suture, follow-up times should encompass clinically significant degradation periods, acknowledging the heterogeneity in results due to varying suture degradation times. The outcome indicator category of SR should include the metric ‘healing progress’.

The study indicates that adverse event occurrence rates exhibited no statistically significant differences compared to control groups in the field of gastrointestinal surgery with a large sample size. Conversely, positive/negative results vary in conclusions in surgical areas with smaller sample sizes. Previous research suggests a significant correlation between sample size and AE occurrence rates [72] Inadequate information in meta-analyses may compromise the reproducibility and representativeness of result, potentially leading to false-negative or false-positive conclusions due to random errors [73] In this study, there was no statistical assessment of whether the sample size in meta-analysis was sufficient for reliable conclusions. Sequential trial analysis, offering statistical evaluations of result reliability, could be beneficial in future studies [74].

This study provides the first comprehensive summary of SRs on BS, outlining AE research findings, current status, and potential applications. However, several limitations should be noted. Due to heterogeneity in AE data and partial overlap among primary studies, quantitative synthesis of the extracted AE data was unfeasible. Additionally, there is no unified classification standard for AEs related to suture applications; Therefore, we proposed a classification framework based on multiple sources, including FDA definitions and complications reported in veterinary suture use. We also recognize that the brand of suture material may significantly impact AE outcomes; however, as SRs rarely provide clear brand-specific data, we were unable to conduct corresponding analyses. We recommend that future SRs pay greater attention to this aspect. In the absence of an authoritative assessment scale for AE entries in SRs, we evaluated relevant items using the harms reporting CONSORT statement for RCTs and SRs [7, 11] Furthermore, all AE outcomes in this study rely on SRs, and we did not independently search for AEs in primary studies, potentially leading to the omission of case reports highlighting rare complications.

## Conclusion

Evidence from SRs has demonstrated advantages as well as certain disadvantages of BSs —such as an increase in the likelihood of suture breakage and wound-related complications. Current evidence remains inadequate and inconsistent, due to lack of definitions for AEs, severity grading, sample size assessments, and details on suture brand and location of use. Our study highlights these methodological shortcomings and provides a structured framework for evaluating AEs in future SRs. Moving forward, rigorously designed, high-quality, and large-sample SRs that incorporate standardized criteria are essential to strengthen the evidence base. Such efforts will support the development of robust guidelines and consensus statements for the safe and effective use of suture-related medical consumables across diverse surgical fields.

## Electronic supplementary material

Below is the link to the electronic supplementary material.

Supplementary Material 1

## Data Availability

No datasets were generated or analysed during the current study.
