# Supplementary material for: Incidence and outcome characteristics of adverse event in surgery: an assessment based on systematic reviews of barbed suture
Source: BMC Med Res Methodol. 2025 Jul 1;25:166. doi: 10.1186/s12874-025-02607-0 (PMC12211956; doi:10.1186/s12874-025-02607-0)

# Appendix S1: Search strategies

**EMBASE SEARCH**

1 exp barbed suture/

2 ((knotless or barbed) and sutur*).tw.

3 quill.tw.

4 Stratafix.tw.

5 V-Loc.tw.

6 1 or 2 or 3 or 4 or 5

7 exp "systematic review"/

8 exp meta analysis/

9 meta analy*.tw.

10 systematic review*.tw

11 7 or 8 or 9 or 10

12 6 and 11

**PubMed SEARCH**

((((((("Sutures"[Mesh]) AND (barbed[Title/Abstract])) OR (("Sutures"[Mesh]) AND (knotless[Title/Abstract]))) OR ((barbed[Title/Abstract] OR knotless[Title/Abstract]) AND (sutur*[Title/Abstract]))) OR (quill[Title/Abstract])) OR (stratafix[Title/Abstract])) OR (v-loc[Title/Abstract])) AND (((("Systematic Review" [Publication Type] OR "Systematic Reviews as Topic"[Mesh]) OR ("Meta-Analysis" [Publication Type] OR "Meta-Analysis as Topic"[Mesh] OR "Network Meta-Analysis"[Mesh])) OR (systematic review*[Title/Abstract])) OR (meta analy*[Title/Abstract]))

**WanFang Data**

(主题:("quill") or 主题:("v-loc") or 主题:("stratafix") or 主题:("薇乐") or 主题:("快翎") or 主题:("鱼骨线") or (主题:("倒刺" or "无结" or "免打结" or "无需打结") and 主题:("线" or "缝合"))) and ((主题:(系统) and 主题:(评价 or 综述)) or 主题:(meta分析) or 主题:(荟萃分析))

**CNKI**

(篇关摘%((倒刺 + 无结 + 免打结 + 无需打结) * (线 + 缝合)) + quill + stratafix + "v-loc" + 快翎 + 薇乐 + 鱼骨线) AND (篇关摘%(系统 * (评价 + 综述)) + 荟萃分析 + meta分析)

**CBM**

#1 "缝合技术"[不加权:扩展] OR "伤口缝合技术"[不加权:扩展]

#2 "缝线"[不加权:扩展]

#3 ( "倒刺"[全部字段:智能] OR "免打结"[全部字段:智能] OR "无结"[全部字段:智能] OR "无需打结"[全部字段:智能])

#4 "线"[全部字段:智能] OR "缝合"[全部字段:智能]

#5 "quill"[全部字段:智能] OR "stratafix"[全部字段:智能] OR "v-loc"[全部字段:智能] OR "快翎"[全部字段:智能] OR "鱼骨线"[全部字段:智能] OR "薇乐"[全部字段:智能]

#6 #1 AND #3

#7 #2 AND #3

#8 #3 AND #4

#9 #5 OR #6 OR #7 OR #8

#10 "系统"[全部字段:智能] AND( "评价"[全部字段:智能] OR "综述"[全部字段:智能])

#11 "meta分析"[不加权:扩展]

#12 "荟萃分析"[全部字段:智能]

#13 "meta分析"[全部字段:智能]

#14 #10 OR #11 OR #12 OR #13

#15 #9 AND #14

**VIP**

任意字段=(倒刺 + 无结 + 免打结 + 无需打结) AND (线 + 缝合) + quill + stratafix + "v-loc" + 快翎 + 鱼骨线 + 薇乐 AND 任意字段=系统 AND (评价 + 综述) + 荟萃分析 + meta分析

# Table S1. Adverse event characteristics assessment items for SR

| Item | Descripter |
| --- | --- |
| 1) Type of indicators in the purpose of SR | Effectiveness only, adverse events only, or both. |
| 2) Scope of concern for AE indicators | Only focus on specific AEs and statistically assess the magnitude of the risk for that event, or explore all possible AEs (describe whether unexpected results were extracted). |
| 3) Types of primary outcomes | Effectiveness only, adverse events only, or both, or no distinction is made. |
| 4) Types of AE outcomes | Aggregated metrics, individual metrics, or both. |
| 5) Definition of the AEs | Definitions are provided for all indicators, or partially provided, or none. |
| 6) Severity grading of AE outcomes | The severity grading of indicators are carried out (and provided classification method), or not. |
| 7) Types of analysis of AE outcome data | Quantitative statistics, qualitative analysis or both, whether to provide the number of events. |
| 8) Evaluation of Optimal Information Sample Size | Yes (and provided the evaluation method), or no. |
| 9) Specify the time frame of surveillance  for AEs | Yes, partially yes, or no. |

# Table S2. Detailed information of each systematic review’s characteristics

| Included study | Published Year | Country | Publication type | Type of surgery | Included primary studies | NO. of subjects （Barbed / Control） | Study Arm | BS brand | Control Arm | Clinical Outcomes | Severity grading of AE outcomes | Follow-up | Definition of AEs |
| --- | --- | --- | --- | --- | --- | --- | --- | --- | --- | --- | --- | --- | --- |
| THA, TJA, TKA, Spinal Surgery | | | | | | | | | | | | | |
| Raja 2022 | 2022 | India | SR & Meta | THR, TKR | 16 RCTs | 1372/1025 | BS | Quill/Stratafix | CS (Including antibacterial suture) | Wound closure time, Cost comparison, Length of stay, Overall complications, Superficial infection, Stitch abscess, Wound discharge, Wound dehiscence, Pinpricks, Broken sutures, ROM, Knee Society score (KSS) | N | 0.5-12 months | N |
| Borzio 2016 | 2016 | USA | SR & Meta | THA, TJA, TKA | 4 RCTs | 290/298 | Directional BS | Quill | CS | Time saving (Mean time for closure), Minor complications (Prominent suture, Superficial infection, Stich abscess, Erythema, or other), Major complications (Deep infections, Pulmonary embolism, Wound dehiscence, Dehiscence of extensor mechanism), Cost saving, Needle sticks, Suture breakage | Y  (minor/ major, according to the definition for each individual study. ) | N | PY |
| Han 2018 | 2018 | China | SR & Meta | THA, TJA, TKA | 5 RCTs | 345/352 | BS | Quill/Stratafix | CS | Wound closure time, Complications (Stitch abscess, Wound dehiscence, Cellulitis, Peri-incisional erythema, Pulmonary embolism), Knee range of motion, Knee Society score, Wound closure cost, Intraoperative events (Suture breakages, Needle stick injuries) | N | 1.5-12 months | N |
| Khlopas 2019 | 2019 | USA | SR | THA, TJA, TKA | 1 RCTs, 5 Retros | 608/653 | BS (BBS) | Quill | Interrupted CS | Closure time, Infection, Postoperative complications, Costs | N | N | N |
| Li 2020 | 2020 | China | SR & Meta | TKA | 6 RCTs | 403/423 | BS | Quill | CS | Wound closure time, Wound closure total cost, Complications (No details), Knee Society scores, Knee range of motion, Suture breakages, Acupuncture injury | N | 1.5-12 months | N |
| Faour 2018 | 2018 | USA | SR | TKA, THA, robot-assisted unicompartmental KA | 5 RCTs, 6 Retros | 1276/1605 | BS | Quill/Stratafix/V-Loc | CS | Wound-related complications (Stitch abscess, Superficial infection, Deep infection, Dehiscence, Prominent suture, Hematoma, Wound leaks), Closure and Operative time, ROM, KSS, Cosmesis and Patient satisfaction | N | N | N |
| Meena 2015 | 2015 | India | SR & Meta | TKA | 1 RCT, 4 Retros | 669/700 | BS | NR | CS | Superficial infection, Deep infection, Wound dehiscence, Arthrofibrosis, Total operative time, Closure time | N | N | N |
| Sun 2021 | 2021 | China | SR & Meta | TKA | 12 RCTs | 771/701 | BS | Quill/Stratafix/V-Loc | CS | Total wound closure time, Wound-related complications (No details), Superficial infection, Stitch abscess, Blister, Ecchymosis, Exudation, Needle prick, Broken sutures, Knee range of movements | N | N | N |
| Xin 2019 | 2019 | China | SR & Meta | TKA | 9 RCTs, 1 Pros, 4 Retros | 1255/1247 | BS | Quill/V-Loc | CS | The total cost for suture, The total time of closure, Knee Society scores, Any complications & Wound complications, Superficial infection & Deep infection, Arthrofibrosis, Wound dehiscence & Stitch abscess, Needle sticks & Suture breakage | N | 1-12 months | N |
| Wei Zhang 2016 | 2016 | China | SR & Meta | Knee Arthroplasty, KA | 4 RCTs, 1 Pros, 5 Retros | 814/915 | BS (BBS & UBS) | Quill/V-Loc | CS (including antibacterial suture) | Wound closure time, Risk of total complications, Major complications and Other complications (Other complications included: Superficial infection, Deep infection, Wound dehiscence, Arthrofibrosis, Hematoma, Suture abscess), Postoperative KSS, Cost | Y  (minor/ major, according to the definition for each individual study. ) | 1.5-12 months | N |
| Ma 2020 | 2020 | China | SR & Meta | TKA | 8 RCTs | 482/502 | BS | NR | CS | Wound closure time, Wound closure total cost, Complications (No details), KSS, KOM, Suture breakages, Acupuncture injury | N | 0.5-12 months | N |
| Xu 2018 | 2018 | China | SR & Meta | TKA | 6 RCTs | 436/434 | BS | NR | Interrupted CS | Suture time, Postoperative complications, Postoperative KSS, Acupuncture injury, Suture breakages | N | N | N |
| Zhiliang Zhang 2019 | 2019 | China | SR & Meta | THA, TKA | 8 RCTs | 476/498 | BS | NR | CS | Incision suture time, Infection, Incision-related complications (No details), KSS, KOM | N | 0.5-12 months | N |
| Wang 2018 | 2018 | China | SR & Meta | TKA | 6 RCTs, 5 Pros/Retros | 588/709 | BS | NR | CS | Joint capsule suture time, HSS at 3 months, Incision complications, Superficial infection, Deep infection, Aseptic redness and swelling (Redness and swelling of incision or Stitch abscess), Incision dehiscence, Suture breakage, Acupuncture injury | N | N | N |
| Zhan 2019 | 2019 | China | SR & Meta | THA, TJA, TKA | 11RCTs | 852/777 | BS | NR | CS | Wound closure time, Wound-related complications (No details), Superficial infection, Stitch abscess, Blister, Ecchymosis, Exudation, Broken sutures, Needle prick, KOM at 6 months, KSS | N | N | N |
| Li 2020 | 2020 | China | SR & Meta | TKA | 8 RCTs, 3 Retros | 1176/1133 | BS | NR | CS | Time for capsule suture, Superficial wound infection, Deep wound infection, Wound dehiscence, Suture breakage, Needlestick, Hospital for Special Surgery (HSS), Knee questionnaire, Functional Scoring System (KSS) | N | N | N |
| Russo 2024 | 2024 | Italy | SR | THA | 5 RCTs, 2 Pros, 2 Retros | 6959 / 6959 | BS (UBS) | NR | CS | Blood Loss, Closure Time, Costs, Complications (wound-related complication, dehiscence, superficial wound infection) | N | 3-6 months | N |
| Pustilnik 2025 | 2025 | Brazil | SR & Meta | Spinal Surgery | 1 RCT, 3 Pros, 2 Retros | 535/700 | BS | NR | CS | Deep infection, Superficial Infection, Total Infection, Hospital stay, Operating room time, Postoperative hematoma, Reintervention Due to Wound Healing Problems, Suturing time, Wound dehiscence/seroma, Total Hospitalization Cost | N | N | N |
| Sarhan 2024 | 2024 | Poland | SR & Meta | Posterior spine surgery | 1 RCT, 1 Pros, 5 Retros | 4240/4405 | BS (BBS; UBS) | Quill/Stratafix | CS | operative time, wound closure time, post-operative wound complications, overall post-operative wound complications, wound infections, dehiscence or seroma, hematoma, hospital stay, reintervention rates | N | 3-15 months | N |
| Cesarean delivery/Laparoscopic hysterectomy/Laparoscopic myomectomy | | | | | | | | | | | | | |
| Agarwal 2021 | 2021 | Canada | SR & Meta | Cesarean delivery | 4 RCTs | 263/197 | BS (BBS) | Quill/Stratafix | CS  (Including antibacterial suture) | Combined postoperative morbidity (Estimated blood loss, Postoperative pain score, Surgical site infections, Postoperative fever, Wound dehiscence, Need for blood transfusion, Intestinal obstruction, and Any other reported complications, Scar integrity), Total surgical time, Uterine incision repair time, Additional hemostatic suture, Hollander cosmesis score | N | Only reported all trials had a short follow-up time after the surgery | N |
| Raischer 2022 | 2022 | Israel | SR & Meta | Cesarean delivery | 3 RCTs, 1 Retros | 1473/1859 | BS | NR | CS  (Including antibacterial suture) | Closure time, Total operation time, Perioperative blood loss, Rate of use of additional hemostatic sutures, Need for blood transfusion, Incidence of postoperative complications, Length of hospitalization | N | N | N |
| Bogliolo 2015 | 2015 | Italy | SR & Meta | Robotic-assisted hysterectomy, Total laparoscopic hysterectomy | 2 RCTs, 7 Retros, 1 Pros, 1 Case control | 673/996 | BS  (BBS&UBS) | Quill/V-Loc | CS | Suturing time, Postoperative bleeding (minor and major), Vaginal Cuff Dehiscence | Y (minor complications described narratively) | N | N |
| Iavazzo 2015 | 2015 | Greece | SR & Meta | Laparoscopic myomectomy, Robotic-assisted total hysterectomy, Total laparoscopic hysterectomy | 3 RCTs, 2 Pros, 10 Retros, 4 Case reports | 828/979 | BS (BBS&UBS) | NR | CS | Duration of Operation, Duration of Suturing, Estimated Blood Loss, Major Bleeding, Vaginal Cuff Dehiscence, Complications | N | 0.5-12 months | Y |
| Gardella 2018 | 2018 | Italy | SR & Meta | Laparoscopic myomectomy | 2 RCTs, 4 Retros, 2 Pros | 639/565 | BS (BBS&UBS) | NR | CS | Blood loss, Postoperative hemoglobin (Hb) drop, Length of hospitalization, Suturing time, Total operative time, Peroperative complications, Surgical difficulty (Visual analogical scale) | N | N | N |
| Uccella 2021 | 2021 | Italy | SR & Meta | Robotic-assisted hysterectomy, Total laparoscopic hysterectomy | 6 RCT, 2 Pros, 11 Retros, 1 Case control | 1108/1097 | BS | NR | CS | Cumulative VCD rate | N | N | Y |
| Zheng 2015 | 2015 | China | SR & Meta | Laparoscopic hysterectomy, Laparoscopic myomectomy | 3 RCTs, 2 Non-RCTs | 116/121 | BS | NR | CS | Suturing time, Operative time, Blood loss, The grade of the surgical difficulty, Postoperative complications | N | N | N |
| Kathopoulis 2024 | 2024 | Greece | SR & Meta | Laparoscopic myomectomy | 4 RCTs, 3 Pros, 9 Retros | 1097/1132 | BS (UBS & BBS) | Quill/Stratafix/V-Loc | CS | Operative time, Suturing time, Estimated blood loss, Hemoglobin concentration change, Blood transfusion rate, Surgical difficulty VAS score, Postoperative pain VAS score, Length of hospital stay, Postoperative complication rate, Postmyomectomy Pregnancy rate, Postmyomectomy Live birth rate, Postmyomectomy Pregnancy complication rate | N | N | N |
| Hafermann 2024 | 2024 | Germany | SR & Meta | Gynecological surgery | 5 RCTs, 7 Pros, 13 Retros | 2298/2154 | BS (UBS) | V-Loc | CS | Operation time, Suture time, Estimated blood loss, Length of stay, Total post-operative complications, Surgical site infections, Granulation tissue formation, Surgical difficulty | N | N | N |
| Yuan Zhang 2016 | 2016 | China | SR & Meta | Laparoscopic myomectomy | 2 RCTs, 2 Retros, 3 Pros | 279/205 | BS | NR | CS | Suturing time, Operative time, Perioperative hemoglobin change, Estimated blood loss, Hospital stay | NA | N | NA |
| Tulandi 2014 | 2014 | Canada | SR & Meta | Laparoscopic hysterectomy, Laparoscopic myomectomy | 3 RCTs, 3 Retros, 1 Pro | 410/524 | BS | NR | CS | Operative time, Suturing time, Degree of surgical difficulty, Estimated blood loss | NA | N | NA |
| Colorectal anastomosis/Gastric bypass/Laparoscopic sleeve gastrectomy/Peptic ulcer repair/Right colectomy | | | | | | | | | | | | | |
| Lin 2019 | 2019 | China | SR & Meta | LRYGB, LSG | 4 RCTs, 2 Pros, 2 Retros | 3286/23699 | BS | Stratafix/V-Loc | CS | Suture time, Operative time, Hospital stay, Postoperative complications (Overall, Bleeding, Stenosis, Leak), Cost | Y (Clavien–Dindo) | 3.8-36 months (median) | N |
| Chaouch 2021 | 2021 | Tunisia | SR & Meta | RYGB, SAGB | 2 RCTs, 4 Pros, 2 CCTs | 2965/23375 | BS | Stratafix/V-Loc | CS | Overall morbidity, Operative time, Bleeding, Anastomotic leakage, Anastomotic stricture, Hospital stay, Cost | N | 1-28.7 months | N |
| Velotti 2022 | 2022 | Italy | SR & Meta | RYGB, Right colectomy, Peptic ulcer repair, MGB/OAGB | 2 RCTs, 3 Pros, 5 Retros, 2 Case control | 3372/23761 | BS | NR | CS | Peri-operative bleedings, Peri-operative leaks, Peri-operative stenosis, Operative time | N | N | N |
| Wiggins 2020 | 2020 | UK | SR & Meta | LRYGB, SAGB, Colorectal anastomosis | 2 RCTs, 2 Pros, 4 Retros, 1 Case control | 3031/23444 | BS | NR | CS | Overall operative time, Anastomosis time, Rates of overall morbidity, Anastomotic leak, Anastomotic bleeding, Anastomotic stricture, Overall hospital stay | N | N | N |
| Ataya 2024 | 2024 | Canada | SR & Meta | Bariatric surgery | 4 RCTs, 2 Pros, 5 Retros | 3516/23926 | BS (UBS) | Stratafix/V-Loc | CS | Operative time, Suturing time, Leak, Bleeding, Stenosis, Bowel obstruction, Hospital stay | N | 3.8-60.35 months (Mean range) | N |
| Delgado 2025 | 2025 | Brazil | SR & Meta | Laparoscopic-Assisted Colorectal Surgery | 4 Observational studies | 143/142 | BS | V-Loc | CS | Operative time, Intraoperative complications, Anastomotic leakage, Clavien–Dindo ≥ III complications | Y (Clavien–Dindo) | N | N |
| Partial nephrectomy | | | | | | | | | | | | | |
| Bertolo 2019 | 2019 | USA | SR & Meta | Minimally invasive partial nephrectomy | 1 RCT, 1 Pros, 8 Retros | 267/300 | BS | Quill/Stratafix/V-Loc | CS | Operative time, Warm ischemia time, Blood losses, Postoperative complications, Transfusions, Urinary leakages | Y (Clavien–Dindo) | N | N |
| Lin 2019 | 2019 | China | SR & Meta | Partial nephrectomy | 3 Pros, 5 Retros | 190/241 | BS (BBS&UBS) | V-Loc | CS | Warm ischemia time, Operative time, Estimated blood loss or Change in hemoglobin level, Perioperative blood transfusion, Changes in renal function, Hospital stay, Postoperative complications | Y (Clavien–Dindo) | 0.25-11 months (median) | N |
| Zhan 2019 | 2019 | China | SR & Meta | Laparoscopic partial nephrectomy | 1 RCTs, 7 Retros | 216/261 | BS | Quill/V-Loc | CS | Warm ischemia time, Perioperative complications, Overall operative time, Estimated blood loss, Length of stay, Change of renal function | Y (Clavien–Dindo) | N | N |
| Huo 2018 | 2018 | China | SR & Meta | Laparoscopic partial nephrectomy | 5 RCTs, 7 Retros, 1 Not specified | 545/554 | BS | NR | CS | Total wound closure time, Warm ischemia time, Operative time, P.E.N.A. score, Postoperative complications, Intraoperative blood loss, Postoperative length of hospital stay | N | 1-48 months | N |
| Minimally invasive pyeloplasty | | | | | | | | | | | | | |
| Anand 2022 | 2022 | India | SR & Meta | Minimally invasive pyeloplasty | 1 Pros, 4 Retros | 60/60 | BS | NR | CS | Operative duration, Redo-pyeloplasty due to recurrent UPJO, Incidence of postoperative complications, Length of hospital stay | N | 3-37 months (Mean/median) | N |
| Radical prostatectomy | | | | | | | | | | | | | |
| Bai 2015 | 2015 | China | SR & Meta | Minimally invasive radical prostatectomy | 3 RCTs, 7 Pros | 378/369 | BS (BBS&UBS) | V-Loc | CS | Pperative time, VUA time, Posterior reconstruction time, Urinary leak and retention, Complications, Estimated blood loss, Continence recovery, Length of hospitalization, Catheterization time, | N | N | N |
| Li 2015 | 2015 | China | SR & Meta | Robot-assisted radical prostatectomy | 3 RCTs, 4 Pros, 2 Retros | 417/369 | BS (UBS ) | NR | CS | Anastomosis time, Operative time, Posterior reconstruction (PR) time, Postoperative leakage (PL) rate, Continence rates, Estimated blood loss (EBL), Length of catheterization (LOC) | N | 3-12 months | N |
| Lin 2017 | 2017 | China | SR & Meta | Minimally invasive radical prostatectomy | 3 RCTs, 9 Pros/Retros | 457/452 | BS | NR | CS | Operative time, Suturing time, Estimated blood loss or Change in hemoglobin level, Length of catheterization, Hospital stay, Postoperative complications, Continence rate | N | 4.1-20 months (mean) | N |
| Barbed pharyngoplasty | | | | | | | | | | | | | |
| Moffa 2023 | 2023 | Italy | SR | Barbed pharyngoplasty | 4 Pros, 1 Retros, 1 Longitudinal, 1 Not specifed | 202 (Not specified) | BS | NR | NR | Snoring Visual Analog Scale (VAS) score, Subjective sleep quality improvement, Complications (partial knot extrusion, mucosal granulomas, bleeding, foreign body sensation) | Y (minor complications described narratively) | 1-9 months | N |
| Moffa 2023 | 2023 | Italy | SR | Barbed pharyngoplasty | 1RCT, 6 Pros, 4 Retros, 1 Pilot longitudinal, 1 Longitudinal, 1 Not specifed | 769 (Not specified) | BS | NR | NR | Intra-operative complication（partial thread extrusion, intra-operative self-limited bleeding, broken needle, intra-operative suture rupture, chipped tooth）; Short-term complication（post-operative pain, partial suture/thread extrusion, temporary velopharyngeal insufficiency, excessive postnasal discharge, mucosal granulomas, anterior pharyngoplasty dehiscence, tonsillar haemorrhage, acute post-operative infection, temporary nasal liquid regurgitation, foreign body sensation, transient dysphagia, post-operative bleeding, post-tonsillectomy haemorrhaging, nasopharyngeal insufficiency, no complications）; Long-term complication（foreign body sensation, dry throat, phlegm in throat, mild to moderate dysphagia, rhinolalia, nose regurgitation, sticky mucus sensation in throat, painful throat sensation）. | Y  (minor/ major, according to the definition for each individual study. ) | 1-41.3 months | N |
| Moffa 2020 | 2020 | Italy | SR | Barbed Pharyngoplasty | 1 RCT, 9 Single-arm studies without a control group, 2 Comparison studies between different techniques of barbed pharyngoplasty | 383/NR | BS (BBS) | NR | NR | Apnea-Hypopnea Index (AHI), Epworth Sleepiness Scale (ESS), Oxygen Desaturation Index (ODI), Snoring Visual Analog Scale (VAS), Minor complications (e.g., Thread extrusion, Knot exposure, Transient dysphagia), No major adverse events reported in most studies | Y (minor/major, according to individual study definition) | 1–12 months | Defined per individual study; includes thread extrusion, granulomas, dehiscence, etc. |
| Saenwandee 2022 | 2022 | Thailand | SR & Meta | Barbed pharyngoplasty | 1 RCT, 6 Pros, 10 Retros, 1 Not specifed, 2 Pilot longitudinal | 762 (Not specified) | BS (NR) | NR | NR | Apnea-hypopnea index (AHI), Oxygen desaturation index (ODI), Lowest oxygen saturation (LSAT), Epworth Sleepiness Scale (ESS), Snoring Visual Analog Scale (VAS), Surgical success rate, Operative time, Pain Visual Analog Scale (VAS), Hospital stay, Time with oxygen saturation < 90%, Postoperative complications, Suture extrusion, Velopharyngeal insufficiency (VPI), Tonsillectomy bleeding, Mucosal granulomas, Anterior pharyngoplasty dehiscence, Dysphagia, Infection, Postnasal discharge, Temporary swallowing problems, Rhinolalia, Foreign body sensation, Dysgeusia, Globus sensation, Intraoperative thread extrusion, Intraoperative bleeding, Broken needle, Suture rupture, Severe bleeding and upper airway obstruction (not observed) | N | 1-26 months (median: 6 months) | N |
| Scar Aesthetics, Cosmetic surgery | | | | | | | | | | | | | |
| Motosko 2018 | 2018 | USA | SR | TKA Abdominoplasties Multiple cosmetic surgeries Cesarean sections Gynecologic procedures Abdominoplasties or reduction mammoplasty | 6 RCTs | 600/545 | BS | Quill/V-Loc | CS | Aesthetic results of wounds, Operating times, Complication rates | N | 1.25-36 months | N |
| Su 2023 | 2023 | China | SR & Meta | Cosmetic surgery (including abdominoplasty, DIEP flap breast reconstruction, body contouring) | 5 RCTs, 2 Pros, 7 Retros | 1132/1127 | BS (BBS & UBS) | Quill/V-Loc | CS | Suture time, Operative time, Hospital stay, Cost, Postoperative complications (wound dehiscence, suture extrusion, incisional infection, seroma, hematoma, delayed wound healing) | Y (Clavien–Dindo) | <1-72 months (Median range ) | N |
| Laparoscopic common bile duct exploration | | | | | | | | | | | | | |
| He 2019 | 2019 | China | SR & Meta | Laparoscopic common bile duct exploration | 3 RCTs, 7 Observations | 416/432 | BS (UBS) | NR | CS  (Including antibacterial suture) | Time for bile duct suturing, Operative time, Length of hospital stay, Incidence of bile leakage | N | N | N |
| Multiple Surgical Field | | | | | | | | | | | | | |
| Lin 2016 | 2016 | China | SR & Meta | Cesarean delivery, Laparoscopic myomectomy, RARP, TKA, Laparoscopic hysterectomy, GB, Comestic surgery, Sacrocolpopexy | 17 RCTs | 994/998 | BS (BBS&UBS) | NR | CS | Suture time, Operative time, Estimated blood loss, Postoperative complications | N | N | N |

THR, Total hip replacement; TKR, Total knee replacement; THA, Total hip arthroplasty; TJA, Total joint arthroplasty; TKA, Total knee arthroplasty; KBS, Knotless barbed suture; BBS, Bidirectional barbed suture; KA, Knee arthroplasty; UBS, Unidirectional barbed suture; VCD, Vaginal cuff dehiscence; LRYGB, Laparoscopic Roux-en-Y gastric bypass; LSG, Laparoscopic sleeve gastrectomy; RYGB, Roux-en-Y gastric bypass; SAGB, Single anastomosis gastric bypass; MGB, Mini Gastric Bypass; OAGB, One Anastomosis Gastric Bypass; MIPN, Minimally invasive partial nephrectomy; NA, Not applicable; PN, Partial nephrectomy; LPN, Laparoscopic partial nephrectomy; RP, Radical prostatectomy; LRP, Laparoscopic radical prostatectomy; RARP, Robot-assisted radical prostatectomy; LCBDE, Laparoscopic common bile duct exploration; GB, Gastric bypass; BS, Barbed suture; SRs, Systematic reviews; RCT, randomized clinical trial; CCT: controlled clinical study; Pros: prospective cohort study; Retros: retrospective study; N: No; Y: Yes; PY: partially yes.

# Table S3. Detailed results of each systematic review’s assessment (AMSTAR-2)

| **Author date** | **1** | **2*** | **3** | **4*** | **5** | **6** | **7*** | **8** | **9*** | **10** | **11*** | **12** | **13*** | **14** | **15*** | **16** | **Overall rating judgement** |
| --- | --- | --- | --- | --- | --- | --- | --- | --- | --- | --- | --- | --- | --- | --- | --- | --- | --- |
| Delgado 2025 | Y | Y | Y | PY | Y | Y | PY | PY | Y | N | Y | Y | Y | Y | Y | Y | Moderate |
| Li 2015 | Y | N | Y | Y | Y | Y | Y | N | Y | N | Y | Y | Y | Y | Y | Y | Low |
| Pustilnik 2025 | Y | Y | Y | PY | Y | Y | PY | PY | Y | N | Y | Y | Y | Y | N | Y | Low |
| Kathopoulis 2024 | Y | Y | Y | PY | Y | Y | Y | PY | Y | N | Y | N | Y | Y | Y | Y | Moderate |
| Sarhan 2024 | Y | Y | Y | Y | Y | Y | PY | Y | Y | Y | Y | Y | Y | Y | Y | Y | High |
| Ataya 2024 | Y | Y | N | PY | Y | Y | PY | PY | Y | N | Y | Y | Y | Y | Y | Y | Moderate |
| Hafermann 2024 | Y | Y | Y | PY | Y | Y | PY | PY | Y | N | Y | Y | Y | Y | Y | Y | Moderate |
| Su 2023 | Y | Y | PY | Y | Y | Y | PY | Y | Y | N | Y | Y | Y | Y | Y | Y | Moderate |
| Moffa 2023 | Y | Y | N | PY | Y | Y | PY | PY | Y | N | No MA | No MA | N | N | N | Y | Critically Low |
| Saenwandee 2022 | Y | Y | N | PY | Y | Y | PY | PY | Y | N | Y | Y | Y | Y | N | Y | Low |
| Anand 2022 | Y | N | N | PY | Y | Y | PY | PY | N | Y | Y | N | N | N | N | Y | Critically Low |
| Velotti 2021 | Y | N | N | PY | N | Y | PY | PY | PY | N | Y | N | N | N | Y | Y | Critically Low |
| Raischer 2022 | Y | Y | Y | PY | Y | Y | PY | PY | Y | N | Y | Y | N | N | N | Y | Critically Low |
| Uccella 2021 | Y | Y | N | Y | N | Y | PY | PY | Y | N | Y | N | N | N | N | Y | Critically Low |
| Agarwal 2021 | Y | Y | Y | Y | Y | Y | PY | PY | Y | N | Y | Y | Y | Y | N | Y | Low |
| Chaouch 2021 | Y | N | Y | Y | Y | Y | PY | Y | Y | N | Y | Y | Y | Y | N | Y | Critically Low |
| Sun 2020 | Y | Y | Y | PY | N | Y | PY | PY | Y | N | N | Y | N | N | N | N | Critically Low |
| Wiggins 2020 | Y | N | N | Y | Y | Y | PY | PY | PY | N | Y | Y | Y | N | Y | N | Low |
| Ma 2020 | Y | N | Y | Y | Y | Y | N | Y | Y | N | Y | Y | Y | Y | Y | Y | Critically Low |
| Moffa 2020 | Y | Y | Y | PY | Y | Y | PY | PY | Y | N | No MA | No MA | N | N | N | Y | Critically Low |
| Li 2020 | Y | N | Y | Y | Y | Y | PY | Y | Y | N | Y | Y | Y | Y | Y | Y | Low |
| Lin 2019 | Y | N | Y | Y | N | Y | PY | Y | Y | Y | Y | N | N | Y | N | Y | Critically Low |
| Xin 2019 | Y | N | Y | PY | N | Y | PY | PY | PY | N | Y | N | N | N | Y | Y | Critically Low |
| Zhang 2019 | Y | N | Y | Y | Y | Y | PY | Y | Y | Y | Y | N | N | Y | Y | Y | Critically Low |
| Khlopas 2019 | N | N | Y | Y | Y | N | N | N | N | N | No MA | No MA | N | N | N | Y | Critically Low |
| Bertolo 2019 | Y | Y | N | Y | Y | Y | N | N | PY | N | N | N | N | Y | N | Y | Critically Low |
| Zhan 2019 | Y | N | Y | PY | Y | Y | PY | PY | Y | Y | Y | N | N | N | N | Y | Critically Low |
| Lin 2019 | Y | N | Y | PY | Y | Y | PY | Y | Y | N | Y | N | N | Y | Y | Y | Critically Low |
| Zhan 2019 | Y | N | Y | PY | Y | Y | PY | PY | Y | N | Y | N | N | Y | Y | Y | Critically Low |
| Faour 2018 | Y | N | Y | PY | N | N | PY | N | N | N | No MA | No MA | N | N | N | Y | Critically Low |
| Han 2018 | Y | N | Y | Y | N | Y | PY | Y | Y | N | Y | N | Y | Y | N | Y | Critically Low |
| Gardella 2018 | Y | N | N | PY | N | N | PY | PY | N | N | Y | N | N | N | N | Y | Critically Low |
| Wang 2018 | Y | N | N | Y | Y | Y | PY | PY | Y | N | Y | Y | Y | Y | N | N | Critically Low |
| Motosko 2018 | Y | N | Y | PY | Y | N | PY | Y | N | N | No MA | No MA | N | Y | N | N | Critically Low |
| Lin 2017 | Y | N | Y | Y | N | Y | PY | Y | Y | N | Y | Y | Y | Y | Y | Y | Low |
| Borzio 2016 | Y | N | Y | Y | N | N | PY | PY | N | N | N | N | N | N | N | Y | Critically Low |
| Zhang 2016 | Y | N | Y | Y | Y | Y | PY | PY | Y | N | Y | Y | Y | Y | N | N | Critically Low |
| Lin 2016 | Y | N | Y | Y | N | Y | PY | PY | Y | N | Y | Y | Y | Y | Y | Y | Low |
| Zhang 2016 | Y | N | Y | PY | Y | Y | PY | PY | Y | N | Y | Y | Y | N | Y | Y | Low |
| Bogliolo 2015 | Y | N | N | PY | N | N | PY | PY | N | Y | Y | N | N | Y | N | Y | Critically Low |
| Iavazzo 2015 | Y | N | N | Y | N | N | PY | Y | N | Y | Y | N | N | N | N | Y | Critically Low |
| Meena 2015 | Y | N | Y | Y | Y | Y | PY | PY | N | N | N | N | N | N | N | Y | Critically Low |
| Bai 2015 | Y | N | Y | Y | Y | Y | PY | PY | N | N | Y | N | Y | Y | N | Y | Critically Low |
| Tulandi 2014 | Y | N | Y | Y | N | N | PY | PY | N | N | Y | N | N | N | N | N | Critically Low |
| Li 2020 | Y | N | Y | N | N | Y | PY | PY | Y | N | Y | N | Y | Y | N | Y | Critically Low |
| Moffa 2023 | Y | Y | N | PY | Y | Y | PY | PY | Y | N | No MA | No MA | N | N | N | Y | Critically Low |
| Raja 2022 | Y | N | Y | Y | Y | Y | PY | Y | Y | N | N | Y | Y | N | N | Y | Critically Low |
| Russo 2024 | Y | Y | N | PY | Y | Y | PY | PY | Y | N | No MA | No MA | Y | N | N | Y | Low |
| He 2019 | Y | N | Y | Y | Y | Y | PY | N | N | N | Y | Y | Y | Y | Y | N | Critically Low |
| Huo 2018 | Y | N | Y | Y | Y | Y | PY | PY | Y | Y | Y | N | N | Y | N | N | Critically Low |
| Zheng 2015 | Y | N | Y | PY | N | Y | N | PY | PY | N | Y | N | Y | N | N | N | Critically Low |
| Xu 2018 | Y | N | Y | PY | Y | Y | PY | PY | Y | N | N | N | N | N | Y | N | Critically Low |

*: critical items; Item 1: study questions and inclusion criteria include PICO; Item 2: Protocol registered before commencement of the review; Item 3: included study design explained; Item 4: comprehensive literature search; Item 5: study selection in duplicate; Item 6: data extraction in duplicate; Item 7: list of excluded studies and justification; Item 8: included study description in detail; Item 9: risk of bias from individual studies being included in the review; Item 10: sources of funding of included studies; Item 11: appropriateness of meta-analytical methods; Item 12: risk of bias impact on meta‐analysis considered; Item 13: consideration of risk of bias when interpreting the results of the review; Item 14: heterogeneity of included studies discussed; Item 15: assessment of presence and likely impact of publication bias; Item: 16: author conflict of interest

# Table S4. Baseline characteristics of SRs

| Characteristics | n | % |
| --- | --- | --- |
| 1) Type of indicators in the purpose of SR (N=52) | | |
| Only efficiency indicators | 2 | 3.8 |
| Only AEs | 1 | 1.9 |
| Efficiency & AEs | 49 | 94.2 |
| 2) Scope of concern for AE indicators (N=50) | | |
| Only focus on specific AEs and statistically assess the magnitude of the risk for that event | 2 | 4.0 |
| Explore all possible AEs | 48 | 96.0 |
| 3) Types of primary outcomes (N=52) | | |
| Only efficiency indicators | 1 | 1.9 |
| Only AEs | 3 | 5.8 |
| Efficiency & AEs | 19 | 36.5 |
| No setting | 29 | 55.8 |
| 4) Types of AE outcomes (N=50) | | |
| Only overall complications | 16 | 32.0 |
| Only specific indicators | 10 | 20.0 |
| Overall complications & specific indicators | 23 | 46.0 |
| 5) Definition of the AEs (N=50) | | |
| Yes | 3 | 6.0 |
| No | 46 | 92.0 |
| Partial Yes | 1 | 2.0 |
| 6) Severity grading of AE outcomes (N=50) | | |
| Clavien-Dindo classification | 6 | 12.0 |
| Self-defined gradings | 6 | 12.0 |
| No severity grading | 38 | 76.0 |
| 7) Types of analysis of AE outcome data (N=50) | | |
| Only qualitative description | 8 | 16.0 |
| Number of events is provided | 8 | 16.0 |
| Number of events was not provided | 0 | 0.0 |
| Only quantitative synthesis | 40 | 80.0 |
| Only effect statistics | 0 | 0.0 |
| Effect statistics & detailed data of AEs all provided | 29 | 58.0 |
| Effect statistics & detailed data of AEs partially provided | 11 | 22.0 |
| Qualitative description & quantitative synthesis | 2 | 4.0 |
| Detailed data of AEs all provided | 2 | 4.0 |
| 8) Evaluation of optimal information sample size (N=50) | | |
| Yes | 0 | 0.0 |
| No | 50 | 100.0 |
| 9) Specify the time frame of surveillance for AEs (N=50) | | |
| Yes | 25 | 50.0 |
| No | 25 | 50.0 |

# Table S5. STROBE Statement—Checklist of items that should be included in reports of *cross-sectional studies*

|  | Item No | Recommendation | Yes/No |
| --- | --- | --- | --- |
| **Title and abstract** | 1 | (*a*) Indicate the study’s design with a commonly used term in the title or the abstract | Yes (p.2, lines 40–42) |
|  |  | (*b*) Provide in the abstract an informative and balanced summary of what was done and what was found | Yes (p.2, lines 43–64) |
| Introduction | | |  |
| Background/rationale | 2 | Explain the scientific background and rationale for the investigation being reported | Yes (p.2-3, lines 69–83) |
| Objectives | 3 | State specific objectives, including any prespecified hypotheses | Yes (p.3, lines 84–87) |
| Methods | | |  |
| Study design | 4 | Present key elements of study design early in the paper | Yes (p.3-4, lines 99–131) |
| Setting | 5 | Describe the setting, locations, and relevant dates, including periods of recruitment, exposure, follow-up, and data collection | Yes (p.3-4, lines 99–111) |
| Participants | 6 | (*a*) Give the eligibility criteria, and the sources and methods of selection of participants | Yes (p.4, lines 112–119) |
| Variables | 7 | Clearly define all outcomes, exposures, predictors, potential confounders, and effect modifiers. Give diagnostic criteria, if applicable | Yes (p.4, lines 120–131) |
| Data sources/ measurement | 8* | For each variable of interest, give sources of data and details of methods of assessment (measurement). Describe comparability of assessment methods if there is more than one group | Yes (p.3, lines 89–98；p.4, 121-131) |
| Bias | 9 | Describe any efforts to address potential sources of bias | Yes (p.3, lines 100–102；p.4, 108-111，121-122，127-128) |
| Study size | 10 | Explain how the study size was arrived at | Not Applicable |
| Quantitative variables | 11 | Explain how quantitative variables were handled in the analyses. If applicable, describe which groupings were chosen and why | Not Applicable |
| Statistical methods | 12 | (*a*) Describe all statistical methods, including those used to control for confounding | Not Applicable |
|  |  | (*b*) Describe any methods used to examine subgroups and interactions | Not Applicable |
|  |  | (*c*) Explain how missing data were addressed | Not Applicable |
|  |  | (*d*) If applicable, describe analytical methods taking account of sampling strategy | Not Applicable |
|  |  | (*e*) Describe any sensitivity analyses | Not Applicable |
| Results | | |  |
| Participants | 13* | (a) Report numbers of individuals at each stage of study—eg numbers potentially eligible, examined for eligibility, confirmed eligible, included in the study, completing follow-up, and analysed | Yes (p.4-5, lines 133–140) |
|  |  | (b) Give reasons for non-participation at each stage | Yes (p.5, lines 140–142) |
|  |  | (c) Consider use of a flow diagram | Yes (p.5, lines 140–142) |
| Descriptive data | 14* | (a) Give characteristics of study participants (eg demographic, clinical, social) and information on exposures and potential confounders | Yes (p.5, lines 142–143) |
|  |  | (b) Indicate number of participants with missing data for each variable of interest | Not Applicable |
| Outcome data | 15* | Report numbers of outcome events or summary measures | Yes (p.6-7, lines 142–166) |
| Main results | 16 | (*a*) Give unadjusted estimates and, if applicable, confounder-adjusted estimates and their precision (eg, 95% confidence interval). Make clear which confounders were adjusted for and why they were included | Not Applicable |
|  |  | (*b*) Report category boundaries when continuous variables were categorized | Not Applicable |
|  |  | (*c*) If relevant, consider translating estimates of relative risk into absolute risk for a meaningful time period | Not Applicable |
| Other analyses | 17 | Report other analyses done—eg analyses of subgroups and interactions, and sensitivity analyses | Yes (p.8, lines 187–192; p.8-9, lines 206-210; p.9, lines 220-223, 232-234; p.10, lines 242-244, 255-258;) |
| Discussion | | |  |
| Key results | 18 | Summarise key results with reference to study objectives | Yes (p.15, lines 274–293) |
| Limitations | 19 | Discuss limitations of the study, taking into account sources of potential bias or imprecision. Discuss both direction and magnitude of any potential bias | Yes (p.17, lines 349–363) |
| Interpretation | 20 | Give a cautious overall interpretation of results considering objectives, limitations, multiplicity of analyses, results from similar studies, and other relevant evidence | Yes (p.15-17) |
| Generalisability | 21 | Discuss the generalisability (external validity) of the study results | Yes (p.15, lines 294–302) |
| Other information | | |  |
| Funding | 22 | Give the source of funding and the role of the funders for the present study and, if applicable, for the original study on which the present article is based | Yes (p.18, lines 387–395) |

# Figure S1. Overall methodological quality of SRs


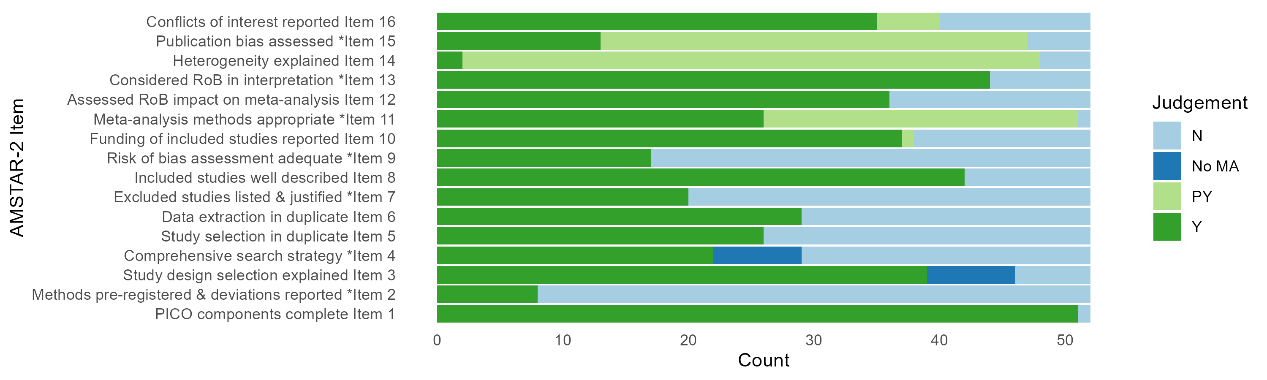


*: AMSTAR 2 critical domains

# Figure S2. Trends in Surgical Fields of Included Systematic Reviews by Published Year


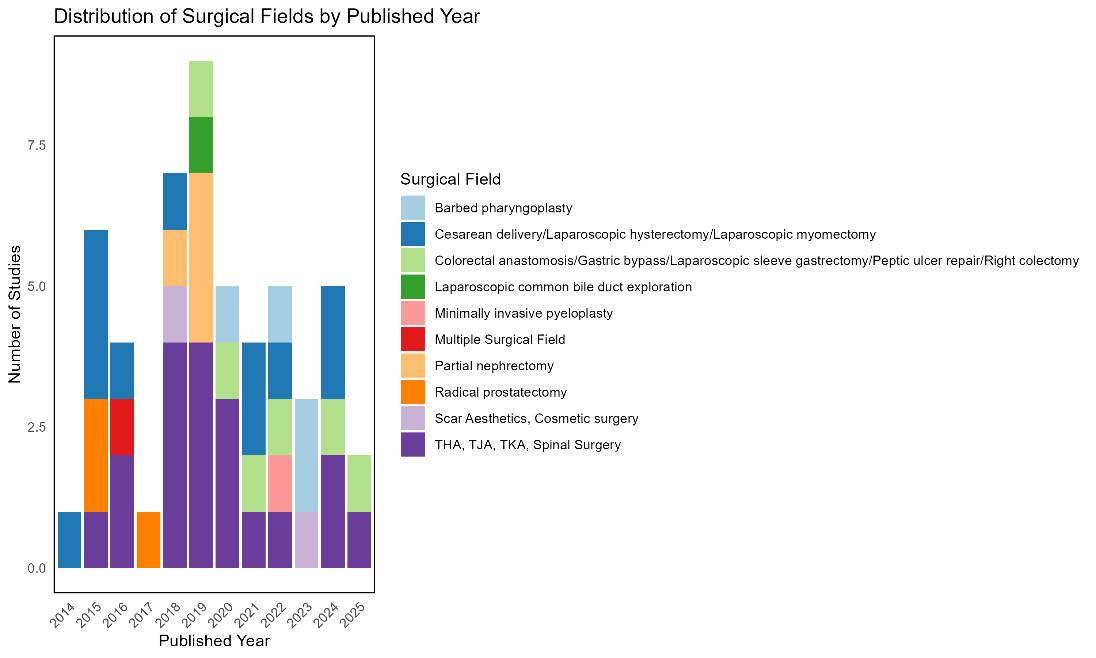

Supplement: Supplementary file 1 — Supplementary Material 1 [file 12874_2025_2607_MOESM1_ESM.docx]
